# Supplementary material for: Violence prevention accelerators for children and adolescents in South Africa: A path analysis using two pooled cohorts
Source: PLoS Med. 2020 Nov 9;17(11):e1003383. doi: 10.1371/journal.pmed.1003383 (PMC7652294; doi:10.1371/journal.pmed.1003383)
Supplement: S1 Table — (DOCX) [file pmed.1003383.s002.docx]

**S1 Table. Baseline characteristics of study participants by loss to follow up.**

|  | Loss to Follow Up | | p-value |
| --- | --- | --- | --- |
|  | Yes  N=223  n (%) | No  N=4811  n (%) |  |
| **Sociodemographic characteristics** | |  |  |
| Province |  |  | <0.001 |
| Eastern Cape | 109 (49) | 1410 (29) |  |
| Western Cape | 98 (44) | 1753 (36) |  |
| Mpumalanga | 16 (7) | 1648 (34) |  |
| Age |  |  | <0.001 |
| Mean (SD) | 14.3 (2.7) | 13.5 (2.4) |  |
| Sex |  |  | 0.537 |
| Female | 131 (59) | 2724 (57) |  |
| Maternal orphan |  |  | 0.698 |
| Yes | 40 (18) | 913 (19) |  |
| Paternal orphan |  |  | 0.485 |
| Yes | 55 (25) | 1090 (23) |  |
| Living with HIV |  |  | <0.001 |
| Yes | 69 (31) | 1042 (22) |  |
| Rural location |  |  | 0.002 |
| Yes | 73 (33) | 2067 (43) |  |
| Informal housing |  |  | 0.49 |
| Yes | 66 (30) | 1322 (28) |  |
| Household size |  |  | 0.882 |
| Mean (SD) | 5.8 (3.1) | 5.7 (2.6) |  |
| **Hypothesised protective factors for violence** | |  |  |
| Positive parenting |  |  | 0.531 |
| Mean (SD) | 12.4 (3.7) | 12.3 (3.7) |  |
| Child monitoring and supervision |  |  | 0.019 |
| Mean (SD) | 9.4 (2.9) | 9.8 (2.6) |  |
| Food security at home |  |  | 0.092 |
| Yes | 185 (83) | 3782 (79) |  |
| Basic economic security at home |  |  | 0.822 |
| Yes | 92 (41) | 1960 (41) |  |
| Free schooling |  |  | 0.687 |
| Yes | 111 (50) | 2338 (49) |  |
| Free school meals |  |  | 0.077 |
| Yes | 183 (82) | 4149 (86) |  |
| **Violence outcomes** | |  |  |
| Sexual abuse |  |  | 0.245 |
| Yes | 5 (2) | 180 (4) |  |
| Transactional sexual exploitation |  |  | 0.289 |
| Yes | 12 (5) | 190 (4) |  |
| Physical abuse |  |  | 0.457 |
| Yes | 71 (32) | 1648 (34) |  |
| Emotional abuse |  |  | 0.022 |
| Yes | 78 (35) | 1343 (28) |  |
| Community violence victimisation |  |  | 0.012 |
| Yes | 110 (49) | 1965 (41) |  |
| Youth lawbreaking |  |  | 0.986 |
| Yes | 60 (27) | 1297 (27) |  |

Data are mean (SD) for continuous variables, and n (%) for categorical variables.
