# Supplementary material for: Violence prevention accelerators for children and adolescents in South Africa: A path analysis using two pooled cohorts
Source: PLoS Med. 2020 Nov 9;17(11):e1003383. doi: 10.1371/journal.pmed.1003383 (PMC7652294; doi:10.1371/journal.pmed.1003383)
Supplement: S2 Table — Data are mean (SD) for continuous variables and n (%) for categorical variables. SD, standard deviation. (DOCX) [file pmed.1003383.s003.docx]

**S2 Table. Baseline characteristics of study participants by province of residence.**

|  | Eastern Cape N=1410  n (%) | Western Cape N=1753  n (%) | Mpumalanga N=1648  n (%) | p-value |
| --- | --- | --- | --- | --- |
| Age |  |  |  | <0.001 |
| mean (SD) | 13.81 (2.95) | 13.42 (2.18) | 13.43 (2.10) |  |
| Sex |  |  |  | 0.096 |
| Female | 798 (57) | 1024 (58) | 902 (55) |  |
| Maternal orphan |  |  |  | <0.001 |
| Yes | 535 (38) | 142 (8) | 236 (14) |  |
| Paternal orphan |  |  |  | <0.001 |
| Yes | 396 (28) | 316 (18) | 378 (23) |  |
| Living with HIV |  |  |  | <0.001 |
| Yes | 995 (71) | 28 (2) | 19 (1) |  |
| Rural location |  |  |  | <0.001 |
| Yes | 386 (27) | 873 (50) | 808 (49) |  |
| Informal housing |  |  |  | <0.001 |
| Yes | 254 (18) | 1003 (57) | 65 (4) |  |
| Household size |  |  |  | <0.001 |
| Mean (SD) | 7.02 (3.09) | 4.63 (1.66) | 5.78 (2.31) |  |
| **Hypothesised protective factors for violence** | |  |  |  |
| Positive parenting |  |  |  | <0.001 |
| Yes | 13.75 (3.10) | 11.08 (3.55) | 12.28 (3.83) |  |
| Child monitoring and supervision |  |  |  | <0.001 |
| Yes | 9.80 (3.13) | 9.23 (2.49) | 10.38 (2.11) |  |
| Food security at home |  |  |  | <0.001 |
| Yes | 1209 (86) | 1264 (72) | 1309 (79) |  |
| Basic economic security at home |  |  |  | <0.001 |
| Yes | 424 (30) | 1198 (68) | 716 (43) |  |
| Free schooling |  |  |  | <0.001 |
| Yes | 760 (54) | 370 (21) | 830 (50) |  |
| Free school meals |  |  |  | <0.001 |
| Yes | 29 (2) | 67 (4) | 84 (5) |  |
| **Violence outcome** | |  |  |  |
| Sexual abuse |  |  |  | <0.001 |
| Yes | 29 (2) | 67 (4) | 84 (5) |  |
| Transactional sexual exploitation |  |  |  | <0.001 |
| Yes | 99 (7) | 52 (3) | 39 (2) |  |
| Physical abuse |  |  |  | <0.001 |
| Yes | 283 (20) | 724 (41) | 641 (39) |  |
| Emotional abuse |  |  |  | <0.001 |
| Yes | 182 (13) | 698 (40) | 463 (28) |  |
| Community violence victimisation |  |  |  | <0.001 |
| Yes | 430 (30) | 1129 (64) | 406 (25) |  |
| Youth lawbreaking |  |  |  | <0.001 |
| Yes | 330 (23) | 468 (27) | 499 (30) |  |
| Data are mean (SD) for continuous variables, and n (%) for categorical variables. | | | | |
