# Supplementary material for: Violence prevention accelerators for children and adolescents in South Africa: A path analysis using two pooled cohorts
Source: PLoS Med. 2020 Nov 9;17(11):e1003383. doi: 10.1371/journal.pmed.1003383 (PMC7652294; doi:10.1371/journal.pmed.1003383)
Supplement: S3 Table — With listwise deletion N = 4,641. CI, confidence interval; OR, odds ratio. (DOCX) [file pmed.1003383.s004.docx]

**S3 Table. Univariable associations between hypothesized protective factors and violence outcomes.**

|  | Boys | | Girls | |
| --- | --- | --- | --- | --- |
|  | OR (95% CI) | p-value | OR (95% CI) | p-value |
| **Sexual abuse** | |  |  |  |
| Positive parenting | 0.98 (0.94; 1.03) | 0.51 | 0.95 (0.92; 0.98) | 0.001 |
| Parental monitoring and supervision | 0.97 (0.91; 1.04) | 0.446 | 0.93 (0.89; 0.97) | 0.002 |
| Food security | 1.17 (0.61; 2.23) | 0.639 | 0.50 (0.34; 0.73) | <0.001 |
| Non-food poverty | 1.53 (0.82; 2.85) | 0.184 | 0.71 (0.43; 1.17) | 0.179 |
| Free schooling | 1.21 (0.67; 2.18) | 0.526 | 0.91 (0.61; 1.35) | 0.647 |
| Free school meals | 0.45 (0.24; 0.83) | 0.012 | 0.65 (0.42; 0.99) | 0.045 |
| **Transactional sex** | |  |  |  |
| Positive parenting | 0.98 (0.95; 1.01) | 0.135 | 0.98 (0.96; 1.00) | 0.086 |
| Parental monitoring and supervision | 0.87 (0.84; 0.91) | <0.001 | 0.88 (0.85; 0.91) | <0.001 |
| Food security | 0.89 (0.61; 1.30) | 0.541 | 0.51 (0.39; 0.67) | <0.001 |
| Non-food poverty | 0.65 (0.41; 1.04) | 0.073 | 0.51 (0.35; 0.75) | 0.001 |
| Free schooling | 0.71 (0.48; 1.03) | 0.074 | 0.32 (0.23; 0.45) | <0.001 |
| Free school meals | 0.47 (0.32; 0.69) | <0.001 | 0.47 (0.36; 0.63) | <0.001 |
| **Physical abuse** | |  |  |  |
| Positive parenting | 0.99 (0.97; 1.01) | 0.194 | 0.98 (0.97; 1.00) | 0.018 |
| Parental monitoring and supervision | 0.99 (0.97; 1.02) | 0.493 | 0.98 (0.96; 1.00) | 0.025 |
| Food security | 0.76 (0.62; 0.92) | 0.006 | 0.72 (0.61; 0.85) | <0.001 |
| Non-food poverty | 1.22 (0.99; 1.51) | 0.065 | 1.07 (0.88; 1.30) | 0.474 |
| Free schooling | 1.24 (1.03; 1.50) | 0.025 | 1.13 (0.96; 1.33) | 0.148 |
| Free school meals | 1.19 (0.93; 1.52) | 0.166 | 1.16 (0.94; 1.42) | 0.16 |
| **Emotional abuse** | |  |  |  |
| Positive parenting | 0.96 (0.95; 0.98) | <0.001 | 0.95 (0.94; 0.96) | <0.001 |
| Parental monitoring and supervision | 0.93 (0.91; 0.96) | <0.001 | 0.93 (0.90; 0.95) | <0.001 |
| Food security | 0.54 (0.43; 0.68) | <0.001 | 0.62 (0.52; 0.75) | <0.001 |
| Non-food poverty | 0.86 (0.66; 1.12) | 0.251 | 0.61 (0.48; 0.78) | <0.001 |
| Free schooling | 1.24 (0.99; 1.56) | 0.06 | 0.93 (0.77; 1.12) | 0.44 |
| Free school meals | 0.85 (0.64; 1.12) | 0.239 | 0.91 (0.73; 1.14) | 0.416 |
| **Community violence victimisation** | |  |  |  |
| Positive parenting | 0.94 (0.92; 0.95) | <0.001 | 0.94 (0.92; 0.95) | <0.001 |
| Parental monitoring and supervision | 0.89 (0.87; 0.91) | <0.001 | 0.90 (0.88; 0.92) | <0.001 |
| Food security | 0.61 (0.50; 0.73) | <0.001 | 0.54 (0.46; 0.63) | <0.001 |
| Non-food poverty | 0.34 (0.27; 0.43) | <0.001 | 0.26 (0.20; 0.33) | <0.001 |
| Free schooling | 1.24 (1.03; 1.49) | 0.021 | 1.55 (1.32; 1.83) | <0.001 |
| Free school meals | 0.99 (0.79; 1.25) | 0.941 | 1.25 (1.02; 1.54) | 0.03 |
| **Youth lawbreaking** | |  |  |  |
| Positive parenting | 0.95 (0.93; 0.96) | <0.001 | 0.95 (0.93; 0.96) | <0.001 |
| Parental monitoring and supervision | 0.90 (0.88; 0.92) | <0.001 | 0.93 (0.90; 0.95) | <0.001 |
| Food security | 0.96 (0.77; 1.18) | 0.674 | 0.79 (0.65; 0.96) | 0.02 |
| Non-food poverty | 0.89 (0.70; 1.12) | 0.318 | 0.68 (0.53; 0.87) | 0.003 |
| Free schooling | 0.76 (0.62; 0.93) | 0.008 | 0.81 (0.66; 0.99) | 0.039 |
| Free school meals | 0.66 (0.52; 0.84) | 0.001 | 0.79 (0.62; 0.99) | 0.043 |

With listwise deletion N = 4641.

Abbreviations: OR, Odds ratio; CI, Confidence interval.
