# Supplementary material for: Violence prevention accelerators for children and adolescents in South Africa: A path analysis using two pooled cohorts
Source: PLoS Med. 2020 Nov 9;17(11):e1003383. doi: 10.1371/journal.pmed.1003383 (PMC7652294; doi:10.1371/journal.pmed.1003383)
Supplement: S4 Table — (DOCX) [file pmed.1003383.s005.docx]

**S4 Table. Sensitivity analysis of adjusted predictions comparing single and multiple outcome modelling approach.**

|  | Boys | | | | Girls | | | |
| --- | --- | --- | --- | --- | --- | --- | --- | --- |
|  | Adjusted Probability, % | | Difference (b-a), percentage points | Ratio (b/a), percent | Adjusted Probability, % | | Difference (b-a), percentage points | Ratio (b/a), percent |
|  | a) Single outcome approach | b) Multiple outcome approach |  |  | a) Single outcome approach | b) Multiple outcome approach |  |  |
| **Sexual abuse** | |  |  |  |  |  |  |  |
| No protective factors | 2.39 | 2.98 | 0.59 | 124.75 | 5.38 | 5.40 | 0.02 | 100.44 |
| All three protective factors | 1.80 | 2.03 | 0.23 | 113.01 | 1.64 | 1.77 | 0.13 | 107.78 |
| **Transactional sexual exploitation** | |  |  |  |  |  |  |  |
| No protective factors | 6.97 | 7.16 | 0.18 | 102.65 | 10.07 | 10.03 | -0.05 | 99.55 |
| All three protective factors | 4.55 | 4.67 | 0.12 | 102.72 | 4.84 | 5.04 | 0.20 | 104.08 |
| **Physical abuse** | |  |  |  |  |  |  |  |
| No protective factors | 37.19 | 36.95 | -0.24 | 99.37 | 38.58 | 38.35 | -0.22 | 99.43 |
| All three protective factors | 25.44 | 26.88 | 1.43 | 105.63 | 23.85 | 24.94 | 1.08 | 104.55 |
| **Emotional abuse** | |  |  |  |  |  |  |  |
| No protective factors | 23.72 | 23.99 | 0.27 | 101.14 | 25.39 | 25.70 | 0.31 | 101.21 |
| All three protective factors | 10.72 | 10.56 | -0.16 | 98.55 | 12.98 | 12.61 | -0.37 | 97.16 |
| **Community violence victimisation** | |  |  |  |  |  |  |  |
| No protective factors | 41.28 | 41.45 | 0.17 | 100.40 | 33.64 | 37.38 | 3.75 | 111.13 |
| All three protective factors | 35.41 | 26.97 | -8.44 | 76.16 | 25.08 | 21.43 | -3.65 | 85.44 |
| **Youth lawbreaking** | |  |  |  |  |  |  |  |
| No protective factors | 22.44 | 22.77 | 0.33 | 101.47 | 18.90 | 19.07 | 0.17 | 100.89 |
| All three protective factors | 14.98 | 13.99 | -0.99 | 93.37 | 11.61 | 10.85 | -0.76 | 93.49 |
