# Supplementary material for: Violence prevention accelerators for children and adolescents in South Africa: A path analysis using two pooled cohorts
Source: PLoS Med. 2020 Nov 9;17(11):e1003383. doi: 10.1371/journal.pmed.1003383 (PMC7652294; doi:10.1371/journal.pmed.1003383)
Supplement: Attachment — Submitted filename: cluver.pdf [file pmed.1003383.s006.pdf]

This paper describes the association between experienced violence and provisions to reduce it in young people in South Africa.

I was asked for a statistical report and I interpret that to include all aspects of the design and conduct of the study.

Points of detail

Page 3 I was confused by the reference to two cohorts immediately followed by three names. How about 'two [...] cohorts spread across three provinces: Mpumalanga, Eastern Cape and Western Cape.'?

Page 4 I have never seen the term sero–assorted before. If it is common in the HIV field then fair enough but if not perhaps re–phrase?

Page 4 This is perhaps more for the editors, but would it be better to have the questionnaires, at least in English, as supplementary material in case the cited site becomes inaccessible?

Page 5 I am not quite sure I grasp how the Benjamini–Hochberg procedure was applied. As I read it there are six equations each containing six predictors of scientific interest. So does the family consist of six values, one for each predictor, across equations? If I am correct perhaps re–phrase? Of course if I have got the meaning all wrong then re–phrasing is even more pressing. I suspect one can decode this from the pattern of p–values in Table 2 but the authors could make life simple for us.

Pages 6 and 7, Table 1 I found this hard to read as I kept losing track of which characteristic was associated with which p-value. Removing the horizontal rules except when they demarcate a new variable would help (that is, leave them before Age, Maternal orphan, ... rather like Figure 1 which is much easier to grasp).

Page 8 Given the absence of strong evidence about interactions with sex is it wise to report these separately for boys and girls? The text is quite hard to read and I wonder whether repeating all the information from the table is a good idea rather than summarising the message from the table in words.

Pages 8 and 9 I am all in favour of absolute risk differences but they do have the disadvantage that the confidence intervals can include impossible values. This is the case for several of these here for instance 5.21 to 1.47 is -3.74 (-5.41 to -2.07) but if you start at 5.21 you cannot go

down 5.41 units. The authors do also present risk ratios which are not immune to similar issues but they do not really give them much prominence.

Page 8 By this point I am afraid I had forgotten why these three were selected. I know it is in the methods but a sentence here to remind us would not be a bad idea.

Page 9, Table 3 Does 'All potential' mean all six potential or all three which were selected? I suspect the latter and so All three might be clearer.

In people as young as this is engaging in transactional sex not a subset of sexual abuse? Or have I mis–understood what transactional sex means?

Summary

Mostly points of detail and requests for clarification.

Michael Dewey
