# Supplementary material for: Violence prevention accelerators for children and adolescents in South Africa: A path analysis using two pooled cohorts
Source: PLoS Med. 2020 Nov 9;17(11):e1003383. doi: 10.1371/journal.pmed.1003383 (PMC7652294; doi:10.1371/journal.pmed.1003383)
Supplement: Attachment — Submitted filename: Response to Reviewers.docx [file pmed.1003383.s007.docx]

Public Library of Science,

Carlyle House,

Carlyle Road,

Cambridge,

CB4 3DN, UK

Dear Editor,

**With reference to: PMEDICINE-D-20-01470R1 “Violence prevention accelerators for children and adolescents in Africa” (Cluver *et al.*).**

Thank you for considering our manuscript for publication in PLOS Medicine. We are grateful for your and the reviewers’ thoughtful, and constructive comments. They have helped to improve our manuscript greatly. We are also grateful for your agreeing to give us more time to complete the revisions that the reviewers requested. The additional time was key for enabling us to do our best to address each comment as thoroughly as possible below, and in the attached manuscript. We feel that this makes the manuscript a lot more complete. We hope that it may now be suitable for publication. However, we would of course be pleased to make further improvements if necessary.

Please let me know if I can assist further. On behalf of all those involved in this work, I look forward to hearing your reply

Yours sincerely,

**Professor Lucie Cluver DPhil (Corresponding Author)**

**Email:** [**lucie.cluver@spi.ox.ac.uk**](mailto:lucie.cluver@spi.ox.ac.uk)**; Telephone: +44(0)7980 856651**

**Section editor comments:**

***Please revise your title according to PLOS Medicine's style, this would normally include some indication of the study design (eg, here, an analysis based on 2 prospective cohorts) at the end of the title after a colon.**

*RESPONSE: Thank you for this. We have revised the title to:“Violence prevention accelerators for children and adolescents: A path analysis using two pooled cohorts in South Africa”*

LINE 1: “Violence prevention accelerators for children and adolescents: A path analysis using two pooled cohorts in South Africa”

***Please structure your abstract using the PLOS Medicine headings (Background, Methods and Findings, Conclusions - "Methods and Findings" is a single subsection). In the last sentence of the Abstract Methods and Findings section, please include a brief comment about any key limitation(s) of the study's methodology.**

*RESPONSE: We have now structured the abstract using the PLOS Medicine headings.*

***In the abstract, the acronym CI is used without having previously defined it (presumably, "confidence interval"). In addition, it would be good to explicitly state what the limits for the CIs are, presumably this is 95% but not always, sometimes 99% is seen.**

*RESPONSE: We have clarified that confidence intervals reported in the abstract are 95% Confidence Intervals and defined the acronym CI at first use.*

LINE 67-68: “Prevalence differences and ratios with 95% confidence intervals (CIs) were also calculated.”

***We'd suggest ensuring that the study is reported according to the STROBE guideline; in which case please include the completed STROBE checklist as Supporting Information. Please add the following statement, or similar, to the Methods: "This study is reported as per the Strengthening the Reporting of Observational Studies in Epidemiology (STROBE) guideline (SChecklist)." The STROBE guideline can be found here:** [**http://www.equator-network.org/reporting-guidelines/strobe/**](http://www.equator-network.org/reporting-guidelines/strobe/)**. When completing the checklist, please use section and paragraph numbers, rather than page numbers.**

*RESPONSE: We have completed* *the STROBE checklist for cohort studies and included it in the supplementary material.*

LINE 156-158: “The study was reported according to the Strengthening the Reporting of Observational Studies in Epidemiology (STROBE) checklist for cohort studies, Table S1.”

****Please clarify if the analysis reported in this paper corresponded to that laid out in a prospectively set-out protocol or analysis plan? Please state this (either way) early in the Methods section.***

***a) If a prospective analysis plan (from your funding proposal, IRB or other ethics committee submission, study protocol, or other planning document written before analyzing the data) was used in designing the study, please include the relevant prospectively written document with your revised manuscript as a Supporting Information file to be published alongside your study, and cite it in the Methods section. A legend for this file should be included at the end of your manuscript.***

***b) If no such document exists, please make sure that the Methods section transparently describes when analyses were planned, and when/why any data-driven changes to analyses took place.***

***c) In either case, changes in the analysis-- including those made in response to peer review comments-- should be identified as such in the Methods section of the paper, with rationale.***

*RESPONSE: Thank you. We have now clarified that although the specific analysis reported in this study was not pre-specified, it followed a pre-specified methodological approach that has been developed to investigate factors associated with multiple SDG outcomes in observational data as part of the “Accelerate Hub”. This approach is laid out in our Open Science Framework webpage that can be accessed at: https://osf.io/n6jy7/?view_only=17f148085fde4b3fb645106c6c6e418b.*

LINE 305-308: “Although the specific analysis reported in this study was not pre-specified, it followed a pre-specified methodological approach that has been developed to investigate factors associated with multiple SDG outcomes in observational data as part of the “Accelerate Hub”. This approach is laid out on the study teams Open Science Framework”

**Reviewer 1 Comments:**

**This paper describes the association between experienced violence and provisions to reduce it in young people in South Africa. I was asked for a statistical report and I interpret that to include all aspects of the design and conduct of the study.**

**Points of detail**

**Page 3 I was confused by the reference to two cohorts immediately followed by three names. How about `two [. . . ] cohorts spread across three provinces: Mpumalanga, Eastern Cape and Western Cape.'?**

*RESPONSE: We have clarified that the study used pooled data from two cohorts spread across three provinces.*

LINE 161-163: “Study data was pooled from two large prospective cohorts (Young Carers, and Mzantsi Wakho) spread across three provinces: Mpumalanga, Eastern Cape and Western Cape.”

**Page 4 I have never seen the term sero-assorted before. If it is common in the HIV field then fair enough but if not perhaps rephrase?**

*RESPONSE: This is a good point. The term is common in the HIV field but not beyond, and so we have changed the wording to:*

LINE 171-172: “Participants included N=1519 children and adolescents in the Eastern Cape province, living with and without HIV.”

**Page 4 This is perhaps more for the editors, but would it be better to have the questionnaires, at least in English, as supplementary material in case the cited site becomes inaccessible?**

*RESPONSE: We are happy to have these all as supplementary material, but there are rather a lot of them, running to over a hundred pages when combined:*

1. *Young Carers Study Child/Adolescent questionnaire Round 1*
2. *Young Carers Study Child/Adolescent questionnaire Round 2*
3. *Mzantsi Wakho Study Child/Adolescent questionnaire Round 1*
4. *Mzantsi Wakho Study Child/Adolescent questionnaire Round 2*
5. *Mzantsi Wakho Study Clinic File data questionnaire Round 1*
6. *Mzantsi Wakho Study Clinic File data questionnaire Round 2*

*The actual questionnaires used with children and adolescents were mainly in Xhosa, Tsonga and Pedi, but we do have English translations that may be better understood by readers. We have provided a link to a website with all questionnaires available there (this is our core team website, has been running for over ten years and is very unlikely to become inaccessible), and please let us know if you would like the full questionnaires as supplementary materials.*

**Page 5 I am not quite sure I grasp how the Benjamini-Hochberg procedure was applied. As I read it there are six equations each containing six predictors of scientic interest. So does the family consist of six values, one for each predictor, across equations? If I am correct perhaps rephrase? Of course if I have got the meaning all wrong then rephrasing is even more pressing. I suspect one can decode this from the pattern of p-values in Table 2 but the authors could make life simple for us.**

*RESPONSE: Thank you for this comment. You have correctly understood how we applied the B-H procedure. We have clarified this in the text.*

LINE 289-293: “Fifth, to account for risk of Type I error from multiple hypothesis testing, we adjusted estimated p-values using the Benjamini-Hochberg procedure specified with a false discovery rate of 0.05. The analysis had six regression models each containing six predictors of scientific interest. Hence, a family of tests consisted of six values, one for each predictor, across regression models.”

**Pages 6 and 7, Table 1 I found this hard to read as I kept losing track of which characteristic was associated with which p-value. Removing the horizontal rules except when they demarcate a new variable would help (that is, leave them before Age, Maternal orphan, . . . rather like Figure 1 which is much easier to grasp).**

*RESPONSE: Apologies if this was unclear. We have removed the horizontal lines in Table 1 to help distinguish which p-values relate to which coefficients.*

**Page 8 Given the absence of strong evidence about interactions with sex is it wise to report these separately for boys and girls? The text is quite hard to read and I wonder whether repeating all the information from the table is a good idea rather than summarising the message from the table in words.**

*RESPONSE: This is helpful – thank you. We have adapted the descriptions of the results in each table with a view to summarising rather than describing each detail. If the reviewer or editors would prefer, we can remove all reported statistics from the text (OR, CIs).*

*Although the identified protective factors did not differ by gender, the global evidence is very strong that experience of violence victimisation and perpetration does differ for adolescent boys and girls. There is an emphasis in much of the literature on the importance of disaggregating by gender in violence focused analyses. And indeed our analyses do show different levels of exposure by gender across different violence types – such as higher rates of sexual abuse and transactional sex experienced by girls. For these reasons, we kept the separate reporting, but have added to the discussion section a much fuller discussion about the similarity of protective factors across sexes.*

LINE 486-495: “Although some differences by sex reflect distinct experiences and prevalence of violence victimisation, there was strong similarity in protective factors across sexes. Food security and improved parenting were shared ‘accelerators’ across girls and boys – suggesting that the same protective factors can be beneficial for both sexes. This is especially important when considering implications of these findings for future interventions. Provisions to support food security and parenting primarily act at a household level, where targeting of one sex can be problematic. Evidence of protective associations for both boys and girls can increase both the value and feasibility of delivering INSPIRE-associated provisions. It is notable that in girls, free schooling was also significantly associated with lower probability of transactional sexual exploitation, and youth lawbreaking.”

**Pages 8 and 9 I am all in favour of absolute risk differences but they do have the disadvantage that the confidence intervals can include impossible values. This is the case for several of these here for instance 5.21 to 1.47 is -3.74 (-5.41 to -2.07) but if you start at 5.21 you cannot go down 5.41 units. The authors do also present risk ratios which are not immune to similar issues but they do not really give them much prominence.**

*RESPONSE: Thank you for raising this important issue. Your comment encouraged us to thoroughly check through the Stata programming syntax used in our analysis. This check revealed a small error in the values specified in calculating adjusted prevalences for violence outcomes. Specifically, the average and maximum value relating to positive parenting had been applied to parental monitoring, and vice versa for parental monitoring and positive parenting. Since correcting this, the confidence intervals do not include impossible values. Any changes to predicted prevalences as a result of this error were tiny and have not affected any of the findings. The necessary adaptations have been made to relevant tables and the results section of the manuscript. Since identifying this error we went through and checked the full syntax again. No other errors were identified. For transparency we have uploaded the revised Stata programming syntax used in the analysis to our Open Science Framework web page available at:*

*https://osf.io/n6jy7/?view_only=17f148085fde4b3fb645106c6c6e418b*

**Page 8 By this point I am afraid I had forgotten why these three were selected. I know it is in the methods but a sentence here to remind us would not be a bad idea.**

*RESPONSE: Thank you for this! We have adjusted this sentence to remind the reader of the source of these selected factors:*

LINE 384-387: “Of the six hypothesised protective factors from the INSPIRE framework, three factors, positive parenting, parental monitoring/supervision, and food security, were significantly associated with lower likelihood of three or more violence outcomes at follow-up for either girls or boys, Table 2.”

**Page 9, Table 3 Does ‘All potential’ mean all six potential or all three which were selected? I suspect the latter and so All three might be clearer.**

*RESPONSE: In Table 3, “All potential” does indeed refer to the three protective factors found to be significantly associated with three or more violence outcomes, namely: positive parenting, parental monitoring and supervision, and food security. We have replaced “All potential” with “All three protective factors.”*

**In people as young as this is engaging in transactional sex not a subset of sexual abuse? Or have I misunderstood what transactional sex means?**

*RESPONSE: This is an excellent point, and there is a substantial debate on this in the literature and in the HIV-field in particular. Transactional sex is defined as sex in exchange for something – money, gifts, school grades, transport, shelter or other kinds of support. Whilst some authors and organisations feel that transactional sex that involves under-18 year olds is child sexual exploitation and therefore sexual abuse, others point to complexities. For example, ages of consent vary by country, and some adolescents are having transactional sex with other adolescents of the same age. There is a qualitative literature from African countries suggesting that adolescent girls engage in transactional sex for luxuries (such as make-up) and that this is an active choice by girls not to have sex without getting something in return (the authors do not agree with this, as there is good quantitative evidence that rates of transactional sex are closely related to poverty, and that the introduction of social protection reduces rates of transactional sex). A well-accepted use of language is to refer to ‘transactional sexual exploitation’ and we have changed this in the manuscript throughout. We hope that this clarifies the important reviewer’s point made here.*

**Reviewer 2 Comments**

**Thank you for the opportunity to review the manuscript titled, "Violence prevention accelerators for children and adolescents in Africa" (PMEDICINE-D-20-01470R1). The manuscript reports on an innovative and compelling approach to informing the potential for policy and programmatic impacts on youth experiences of violence. The study has the potential to make significant contributions to the literature and address key questions about effective strategies to improve youth well-being and prevent violence. I recommend acceptance of the paper and note some points for the authors to consider in a revision.**

**1. Although WHO is the publisher of INSPIRE, WHO was not the sole institution to develop and release the technical package. The development was co-led by CDC and WHO, and all of the signatory agencies listed in INSPIRE co-released it, including UNICEF, PEPFAR, USAID, the World Bank, and others. This is one of the key hallmarks of INSPIRE, that it has been co-endorsed and co-released by multiple signatory agencies, allowing for consensus among key institutions in the most effective strategies to address violence against children (page 3).**

*RESPONSE: Thank you for this – you are absolutely correct here. This information was there, but buried at the end of Figure 1. We have now adapted the abstract and introduction (see below). We have also highlighted the partners in INSPIRE by moving these to the top of Figure 1.*

LINE 49-50: “The INSPIRE framework was developed by 10 global agencies as the first global package for preventing and responding to violence against children.”

LINE 100-103: “This has led to global commitments within the Sustainable Development Goals (SDGs) to prevent and respond to violence against children, and to the development led by the World Health Organization (WHO) in 2016 of a set of seven strategies for achieving this – INSPIRE.”

LINE 104-109: “INSPIRE was launched alongside the Global Partnership to End Violence Against Children by the WHO, United States Centers for Disease Control (CDC), the Pan American Health Organisation (PAHO), Presidents Emergency Plan For AIDS Relief (PEPFAR), Together for Girls, United Nations International Children’s Fund (UNICEF), United Nations Office on Drugs and Crime (UNODC), United States Agency for International Development (USAID), and the World Bank.”

**2. Though the content of INSPIRE is primarily focused on prevention, there are also strategies (Response and support services) and programs directed at services for children who have experienced violence. I recommend revising the introduction to use language inclusive of both prevention and response, in places where only prevention of violence is noted (page 3).**

*RESPONSE: Thank you – this is a good point and we have made these revisions throughout the abstract and introduction (examples below). It was with real sadness that we saw in our analyses how few children or adolescents actually accessed any kind of response and support services at all.*

LINE 49-50: “The INSPIRE framework was developed by 10 global agencies as the first global package for preventing and responding to violence against children.”

LINE 95-96: “We are at a pivotal moment of opportunity and risk in preventing and responding to violence against children.”

LINE 112-114: “These strategies are based on reviews of all effective interventions in the growing evidence base for prevention and response to violence against children.”

**3. I recommend including a bit more detail on the data collection procedures for the two studies. Were the questionnaires self-administered? What method of administration was used (paper and pencil, tablet, interview, etc.)? (Page 4)**

*RESPONSE: We agree, and have added further detail on how interviews were conducted.*

LINE 177-181: “For Young Carers, interviews were administered using self-interviewing on paper forms, whereas for Mzantsi Wakho, interviews were administered using audio mobile-assisted self-interviewing on electronic tablets. All interviews were assisted by trained local interviewers, and the level of assistance was adjusted according to the age and literacy level of participants.”

**4. From the description of the measures, it is not clear which are dichotomous/categorical and which are continuous. I checked the questionnaire on the Young Carers website, and given some of the measures use multiple items it is not possible to determine how the responses were coded and used. I recommend including more detail on how the specific outcomes and hypothesized violence prevention provisions were coded for the analyses (page 5).**

*RESPONSE: This is an important clarification. We have clearly reported which variables were binary indicators and which were continuous. We have also added further detail on which questions or items were drawn from relevant instruments for each of the measures used in the analysis. We think this will greatly help the replicability of this study.*

LINE 218-220: “For the analysis, all six outcomes were coded by collapsing responses to multiple self-reported items into binary indicators of any exposure.”

LINE 245-262: “We measured i) Positive parenting/caregiving using a continuous sum of items 2, 13, 16, and 27 from the child form of the Alabama Parenting Questionnaire, which include warmth and praise from a primary caregiver (Range: 0; 16); Given high rates of informal fostering in Southern Africa, we note that ‘parents’ were defined as any primary caregiver and there was no requirement for a biological relationship; ii) Parental/caregiver monitoring and supervision using a continuous sum of items 10, 17, and 19, from the child form of the Alabama Parenting Questionnaire, which include setting rules about coming home in evenings and knowing who an adolescent is friends with (Range: 0; 12); iii) Food security at home as a binary indicator of six or seven days in the past week with enough food in the home; iv) Basic economic security at home as a binary indicator of access to all six of the top socially perceived necessities for youth in the South African Social Attitudes Survey: a doctor when needed, school uniform, basic clothing, toiletries to wash, school equipment, and a pair of shoes; v) Free schooling as a binary indicator of enrolment in a no-fees school, or fee exemption; vi) Free school meals as a binary indicator of daily free lunches or breakfast at school. Exposure to these provisions was measured as consistent reporting at both baseline and follow-up, based on evidence that provisions need to be predictable and sustained in order to protect children and adolescents in high-risk settings.”

**5. The overall pattern of findings suggests that there are more similarities in "accelerator" effects across boys and girls than differences. I think this is a critical point that should be clearly noted in the discussion. However, I found this point to be less prominent. The first paragraph notes that parenting and food security were both accelerators. Based on the lack of significant differences in the gender interaction term it seems to be worth noting more clearly that the differences were in fact few (page 10).**

*RESPONSE: This is another important point, and one that was also made by a previous reviewer. We have expanded this section of the Discussion to make the point more distinct and prominent.*

LINE 486-495: “Although some differences by sex reflect distinct experiences and prevalence of violence victimisation, there was strong similarity in protective factors across sexes. Food security and improved parenting were shared ‘accelerators’ across girls and boys – suggesting that the same protective factors can be beneficial for both sexes. This is especially important when considering implications of these findings for future interventions. Provisions to support food security and parenting primarily act at a household level, where targeting of one sex can be problematic. Evidence of protective associations for both boys and girls can increase both the value and feasibility of delivering INSPIRE-associated provisions. It is notable that in girls, free schooling was also significantly associated with lower probability of transactional sexual exploitation, and youth lawbreaking.”

**Reviewer 3 Comments**

**I think that this complex analysis is very valuable to the field  and worthy of publication.  It is valuable to understand which protective factors are protective for multiple violence outcomes and how that can be leveraged to inform violence prevention programming and policy, particularly in low and middle income settings. I have some fairly minor questions/comments/suggestions for consideration below.**

**1. There are several references to "gender", when I think what is actually meant is biological sex in both the narrative (methods and results) and in the tables. I recommend changing "gender" to "sex".**

*RESPONSE: We have updated all references to “gender”, where biological “sex” was actually implied.*

**2. The rates of sexual abuse and of transactional sex seem fairly low compared to other global surveys (e.g. VACS) and other literature from South Africa. This may be because of the young age of the sample, but it might be helpful to include the actual measures. For example, under sexual abuse in the methods section the sexual abuse measure is described as "two items from the Sexual Victimization module of the Juvenile Victimization Questionnaire". It would be helpful to include what those two measures are.**

*RESPONSE: Thank you for suggesting this. We have been much clearer in our description of how all of our outcomes were measured and how all protective factors were measured.*

LINE 220-222: “Sexual abuse measured as reporting either sexual assault and/or completed rape in the last year using items from the Sexual Victimization module of the Juvenile Victimization Questionnaire.”

**3. My largest comment is in the conceptualization of protective factors (self-reported on questionnaires) as being accelerators of INSPIRE. Based on my reading of the methods section, these study populations did not receive any violence interventions. Rather, they (general population youth) were asked about their experiences with violence victimization and their experience with various protective factors. These protective factors could have been resultant from interventions or not- we do not know because that was not assessed as far as I can gather. Therefore, I am struggling a little bit with the jump from showing in analysis that certain types of protective factors are protective for multiple types of violence to saying that these protective factors are therefore INSPIRE accelerators. In other words, I don't think that we can assume that because something was protective against violence outcomes means that it can therefore be conceptualized as an accelerator for prevention interventions. I actually re-read the methods section several times to understand if there was an intervention, but the way I read the methods section there was no intervention of positive parenting, supervision or food security- these were just self-reported. So I think there might need to be some additional explanation in the discussion section as to why the authors believe that evidence of existing protective factors (without known intervention) having an impact on violence outcomes in the general population can therefore be justification for assuming that interventions that target those protective factors will therefore have the same multiplicative effect on violence outcomes. I also found the term "provisions" to be a bit broad and recommend a different term such as "protective factors" or a similar term.**

*RESPONSE: Thank you for this really valuable comment. You are correct that the study evaluated the association between self-reported protective factors and multiple outcomes. Furthermore, we completely agree with you that there is a leap to assume that identified protective factors may be INSPIRE accelerators. We have now referred to protective factors, and have highlighted at beginning of the methods as well as at several points in the discussion that we investigated the association between intermediary protective factors and violence outcomes, not interventions and violence outcomes. We have kept some reference to ‘accelerators’ as this is a core concept, but have been much more careful not to make a suggestion of causality. We have entirely re-written the discussion section to add a detailed discussion of both the lack of capacity to assume causality or interventions from this data, and also around the possible links between protective factors and interventions targeting these.*

LINE 149-153: “We use secondary data with self-reported measures to examine how variation in protective factors between individuals is associated with multiple forms of violence. All of the protective factors examined are possible targets of INSPIRE interventions. Identifying which protective factors predict lower rates of violence is intended to inform efforts to identify and prioritise the most efficient sub-set of interventions.”

LINE 496-504: “To our knowledge, this study provides the first evidence on which protective factors may be targeted to achieve improvements across diverse violence types simultaneously. Whilst desirable, it would have been too costly to test all protective factors, and their various combinations, using experimental methods. Instead, we took advantage of observed variation between individuals to provide critical guidance on which protective factors could be considered as effective targets for INSPIRE provisions. A drawback of this observational approach is that it is unable to say whether identified relationships between self-reported protective factors and violence outcomes are causal.”

LINE 553-557: “This study provides some of the first evidence on which protective factors could be effective targets for interventions aiming to address multiple violence outcomes simultaneously. Each of the self-reported protective factors found to be associated with multiple violence outcomes have corresponding interventions with randomised evidence of effectiveness in low- and middle-income countries.”
