# Supplementary material for: Violence prevention accelerators for children and adolescents in South Africa: A path analysis using two pooled cohorts
Source: PLoS Med. 2020 Nov 9;17(11):e1003383. doi: 10.1371/journal.pmed.1003383 (PMC7652294; doi:10.1371/journal.pmed.1003383)
Supplement: Attachment — Submitted filename: Response to Reviewers.docx [file pmed.1003383.s008.docx]

Public Library of Science,

Carlyle House,

Carlyle Road,

Cambridge,

CB4 3DN, UK

Dear Editor,

**With reference to: PMEDICINE-D-20-01470R2 “Violence prevention accelerators for children and adolescents: A path analysis using two pooled cohorts in South Africa” (Cluver *et al.*).**

Thank you for planning to accept our manuscript for publication in PLOS Medicine. The peer review process has greatly improved the manuscript, and we are grateful for your patience in this. In the latest revision, we have addressed all of your editorial requests. After completing the required revisions to the abstract, the final wordcount is over the limit of 500 words.

We feel that the revised manuscript is clearer and hope that it may now be suitable for publication. We would of course be pleased to make further changes or improvements if necessary.

Please let me know if I can assist further. On behalf of all those involved in this work, I look forward to hearing your reply.

Yours sincerely,

**Professor Lucie Cluver DPhil (Corresponding Author)**

**Email:** [**lucie.cluver@spi.ox.ac.uk**](mailto:lucie.cluver@spi.ox.ac.uk)**; Telephone: +44(0)7980 856651**

**Section editor comments:**

**1. Title: Please amend to: “Violence prevention accelerators for children and adolescents in South Africa: a path analysis using two pooled cohorts”**

*RESPONSE: Thank you for this. We have revised the title to:“Violence prevention accelerators for children and adolescents in South Africa: a path analysis using two pooled cohorts”*

**2. Abstract:
a. Please include the cohort recruitment dates.
b. Please include summary cohort demographics, eg. age, sex, n and % lost to follow-up.
c. Please quantify all results with p values.
d. Please replace “95% CIs” with “95% CI”
e. Line 71: "…predicted to significantly lower the prevalence…" should become "…was associated with a lower prevalence..." or similar
f. Please present the results for boys and sexual abuse prevalence, to match results presented for girls; mention whether or not significant.
g. In the last sentence of the Methods and Findings subsection, please include key limitation(s) of the study's methodology.
h. Conclusion: Please start this section with a summary of your findings, eg. “In this cohort study, we found that…”.
i. Avoid the use of causal language here and throughout the manuscript, eg. “may be effective” (line 87); use “may be associated with” or similar instead.**

*RESPONSE: We have adapted our abstract to include these points. For point “e.”, the estimates referred to in the abstract are adjusted prevalence estimates. To clarify this, we have been more explicit with our description of them. We have highlighted our responses to some of the more specific points below.*

a. “We pooled data from two prospective South African adolescent cohorts including Young Carers (2010-2012), and Mzantsi Wakho (2014-2017).”

b. “The sample mean age was 13.54 years and 56.62% were female. There was 4% loss to follow up.”

e. “For girls, the adjusted prevalence of violence outcomes was estimated to be lower if all three of these factors were present, as compared to none of them…”

“For boys, the adjusted prevalence of violence outcomes was also estimated to be lower if all three factors were present, as compared to none of them…”

g. “Key limitations were risk of residual confounding and not having information on protective factors related to all seven INSPIRE strategies.”

h. “In this cohort study, we found that positive and supervisory caregiving, and food security at home are associated with reduced risk of multiple forms of violence against children.”

**3. At this stage, we ask that you include a short, non-technical Author Summary of your research to make findings accessible to a wide audience that includes both scientists and non-scientists. The Author Summary should immediately follow the Abstract in your revised manuscript. This text is subject to editorial change and should be distinct from the scientific abstract. Please see our author guidelines for more information: https://journals.plos.org/plosmedicine/s/revising-your-manuscript#loc-author-summary.**

*RESPONSE: We have included a short non-technical author summary in the revised manuscript.*

**4. Methods:
a. Please give exact cohort recruitment dates, including day and month.
b. Please specify whether informed consent was written or oral.
c. Please provide more direct URL links to the participant questionnaires in all languages, or provide them as Supporting Information file(s).
d. Please provide more information on the pilot testing of questionnaires (line 209) in a Supporting Information file or cite published work, as appropriate.
e. Please state whether ethics approval was required or waived for this analysis.**

*RESPONSE: We have clarified each of the points above.*

a. “The Young Carers recruitment took place between 1^st^ of January 2010 and 7^th^ of December 2011, with follow-up between 5^th^ of January 2011 and 15^th^ of December 2012.”

“The Mzantsi Wakho (‘Our South Africa’) recruitment took place between 4th of March 2014 and 25^th^ of September 2015, with follow-up between 10^th^ of November 2015 and 5^th^ of April 2017.”

b. “Both studies obtained written consent from all adolescents and their primary caregivers at both baseline and follow up. Due to varying levels of literacy, informed consent information was read aloud to potential participants.”

c. “All questionnaires are available at [www.youngcarers.org.za](http://www.youngcarers.org.za): Young Carers baseline [12] and follow-up [13]; and Mzantsi Wakho baseline [14] and follow-up [15].

d. “Young Carers, and Mzantsi Wakho questionnaires were piloted with 20, and 25 adolescents from local areas involved in the respective studies. All comments were taken into consideration and errors amended.”

e. “Ethical approvals were gained for Young Carers and Mzantsi Wakho from the University of Oxford (SSD/CUREC2/11-40; SSD/CUREC2/12-21), and University of Cape Town (REC REF: CSSR 389/2009; REC REF CSSR 2013/04) and provincial South African Departments of Health, Basic Education and Social Development. In both cases, ethical approval included examination of predictors of violence with collected data.”

**5. When completing the STROBE checklist, please use section and paragraph numbers, rather than page numbers. Please upload the checklist as a separate file named ‘S1 Checklist’.**

*RESPONSE: Thank you for this clarification. We have completed the STROBE checklist using section and paragraph numbers, and uploaded it as a separate file*

**6. Results: Please quantify all results with p values.**

*RESPONSE: We have quantified all results in the text with p values.*

**7. Please avoid causal language, eg. “may protect against” (line 480); your study can only show association.**

*RESPONSE: Thank you for pointing this out. We have removed any causal language from the manuscript.*

**8. Some of the language is a little extreme (e.g., "groundbreaking in vision and impact" at line 574; "superb" at line 586) – please tone down as appropriate.**

*RESPONSE: We have toned down our language appropriately.*

**9. Please provide full access details (eg. DOI or URL) for references 3, 5, 6, 8, 12, 13, 24, 15, 18, 27, 33, 37, and 40. Re: references 27, 33 listed as forthcoming/in press, papers cannot be listed in the reference list until they have been accepted for publication or are otherwise publicly accessible (for example, in a preprint archive). The information may be cited in the text as a personal communication with the author if the author provides written permission to be named. Alternatively, please provide a different appropriate reference.**

*RESPONSE: We have provided full access details for all references. In the case of references previously listed as forthcoming, they were all published during the handling of our first resubmission.*
